# A protocol for neoWONDER: Neonatal whole population data linkage to improve long-term health and wellbeing of preterm and sick babies

**DOI:** 10.1371/journal.pone.0305113

**Published:** 2024-07-16

**Authors:** Emily van Blankenstein, Alice Aveline, Cheryl Battersby

**Affiliations:** 1 School of Public Health, Imperial College London, London, United Kingdom; 2 Centre for Paediatrics and Child Health, Imperial College London, London, United Kingdom; PLOS: Public Library of Science, UNITED KINGDOM OF GREAT BRITAIN AND NORTHERN IRELAND

## Abstract

**Introduction:**

Early-life medical and surgical interventions in babies born preterm and/or with surgical conditions influence later life health and educational outcomes. Obtaining long-term outcomes post-discharge to evaluate the impact of interventions is complex, expensive, and burdensome to families. Linkage of routinely collected data offers a feasible and cost-effective solution. The NeoWONDER research programme aims to describe the short and long-term health and educational outcomes for babies born preterm and/or with surgical conditions and evaluate the impact of neonatal care and interventions on later health and educational outcomes.

**Methods and analysis:**

We will include babies who received care in neonatal units in England and Wales, born between 2007–2020 with a gestational age below 32 weeks (approximately 100,000), and/or born between 2012–2020 (all gestations) with any of six surgical conditions: necrotising enterocolitis, Hirschsprung’s disease, gastroschisis, oesophageal atresia, congenital diaphragmatic hernia, and posterior urethral valves (approximately 8,000). A detailed list of surgical condition codes is shown in S3 File. We will obtain long-term health and education outcomes through linkage of the National Neonatal Research Database, which contains routine data for all babies admitted to NHS neonatal units, to other existing health and educational datasets. For England, these are: Hospital Episode Statistics, the Office for National Statistics, Mental Health Services Dataset, Paediatric Intensive Care Audit Network, National Pupil Database; and for Wales, the Secure Anonymised Information Linkage databank. Analysis will be undertaken on de-identified linked datasets. Outcomes of interest for health include mortality, hospital admissions, diagnoses indicative of neurodisability and/or chronic illness, health care utilisation; and for education are attainment (using national curriculum assessments), school absence and special educational needs status.

## Introduction

Babies born very preterm (below 32 weeks gestation) represent 2–3% of all UK births (around 8,000 each year). Survival of the most premature babies born before 26 weeks has improved from 40% in 1995 to 56% in 2012 [1], but rates of disability remain unchanged [2]. Very preterm babies are at risk of life-long complications that affect their physical and mental health and their need for health and social care [3]. Cognitive impairment is the most prevalent disability among very preterm babies and contributes to poor educational attainment. Two-thirds require educational support [4]; 23% have mental health problems such as autism spectrum disorder, attention deficit, hyperactivity and emotional disorders [5]. There is a high risk of rehospitalisation and mortality in infancy [6]; asthma and wheezing are highly prevalent [7]. In later life, there are lower rates of employment, income, and self-esteem, as well as higher risk of type 2 diabetes and cardiometabolic problems [8]. The societal cost in England to age 18 is estimated to be around £2.5 billion [5–9].

Reducing complications from preterm birth will improve the life-long health and wellbeing of those born preterm, their families and benefit the wider society by reducing demands on public services. However, no information on long-term outcomes has been available for very preterm babies born in the last decade in the UK to answer the question, ‘*What neonatal interventions or factors post-discharge modify long-term outcomes’*? As survival continues to ameliorate, improving long-term outcomes is a national priority. To do this, long-term data are needed to evaluate the impact of neonatal interventions and inform strategies to improve outcomes through child and in adulthood [10].

There is increasing evidence suggesting that children with chronic health conditions have worse long-term outcomes, including educational attainment [11]. Long-term outcomes for babies with surgical conditions are less well characterised. Major operations performed in the neonatal period or early infancy may have life-long consequences for children [12, 13]. Many children who undergo surgery at this stage in life develop impaired cardiac, neurological, respiratory, gastrointestinal or bladder function, have a life-long requirement for engagement with healthcare, and report lower overall quality of life than their unaffected peers [14–16]. Some of this impact is due to the underlying condition that necessitated surgery, and some due to the interventions required. Given the overall impact of requiring surgery, children may also have lower educational attainment than children who do not require surgery.

This research will benefit children born preterm and/or with surgical conditions by establishing a sustainable approach to the identification of modifiable factors that influence long-term health and developmental outcomes. By demonstrating feasibility of this cost-efficient data linkage approach in the preterm and surgical population, more clinical questions can be addressed at greater pace, to benefit more patients. Demonstrating proof of concept in this exemplar preterm and surgical population will support the continual linkage for future cohorts with complex conditions. Moreover, information on long-term outcomes will support counselling of families, decision-making, and inform future research and public policies to benefit patients and families. This research will also improve the effectiveness and safety of health services by surveillance of long-term harm and benefits. Describing and quantifying long-term outcomes will improve care services by informing the planning of health, community and educational services to meet local needs.

The cost and complexity of obtaining long-term outcome data mean we do not know, at population level, the longer-term outcomes for the 80,000 babies born very preterm in the UK over the last decade. Cochrane recognises the lack of long-term outcomes in randomised controlled trials as a knowledge gap [17]. A search for ongoing preterm trials on the ISRCTN registry yielded 56 studies; none were powered on long-term outcomes [18]. In a systematic review, over half of 76 neonatal trials did not report neurodevelopmental outcomes [19]. Most reported outcomes are short-term (before discharge), which are poor predictors of longer-term functional outcomes [20]. Lack of long-term data hinders the evaluation of meaningful benefit for important functional outcomes. Interventions in the neonatal period may cause inadvertent harm to patients and this harm may only be apparent long after traditional neonatal follow-up finishes. For example, antibiotics given to women in preterm labour increased the risk of cerebral palsy at 7 years [21].

Obtaining long-term data using consent-based cohort studies is complex and expensive, with high attrition over time, limiting the generalisability of findings to a whole population. Another major drawback of opt-in consent based studies, is that seldom heard groups, including those whose English is not their first language, may not participate in opt-in studies. These under-studied groups may represent the segments of the population where long-term conditions have the greatest impact, because of intersectionality with discrimination and poverty.

The UK EPICure studies followed up babies born before 26 weeks in 1995 and 2006. 92% were assessed at 2.5 years and 71% at 11 years in EPICure 1 (283). Those lost to follow-up were more likely to have a non-white ethnic origin, unemployed parents and cognitive impairment. 55% were followed up at 3 years in EPICure 2 (576). As survival improves and numbers rise, these studies are unfeasible and overburdensome for families. Recently, the

US National Children’s Study and the UK Early Life Study were both abandoned due to slow recruitment, resulting in a waste of US $1.2 billion and £9 million.

This study will address these problems by obtaining long-term outcomes through linkage of routine data sources. We will link an established source of routine data on all babies admitted to NHS neonatal units, the National Neonatal Research Database (NNRD), with other routine health, educational and environmental datasets in England and Wales. Further detail on the NNRD can be found in S2 File.

## Methods and analysis

### Aims

1. To describe the long-term health and education outcomes for very preterm-born children born and cared for in England and/or Wales, and children of any gestation with a specified surgical condition cared for in England, and examine factors influencing these outcomes. The specified surgical conditions are: necrotising enterocolitis, Hirschsprung’s disease, gastroschisis, oesophageal atresia, congenital diaphragmatic hernia, and posterior urethral valves
2. To evaluate the impact of neonatal interventions on the later health and educational outcomes of very preterm-born children using an exemplar intervention: fortification of human breast milk

### Patient and public involvement (PPI)

Extensive PPI work was undertaken to inform study design. A mixed methods study was undertaken to explore the views of parents, adults born preterm and health and education professionals on data linkage through focus groups, a large national survey and interviews. This study found support for data linkage with opt-out consent, including the temporary use of identifiers, as a means to carry out research on long-term outcomes [21].

### Inclusion and exclusion criteria

Eligible babies will be identified from the NNRD. They will include those born between 1^st^ Jan 2007 and 31^st^ December 2020:

1. Born in England or Wales, cared for in an English and/or Welsh neonatal unit, with a recorded gestational age at birth below 32 weeks
2. Born in England and cared for in an English neonatal unit, and received surgery with confirmed diagnosis of at least one of 6 conditions: necrotising enterocolitis, Hirschsprung’s disease, gastroschisis, oesophageal atresia, congenital diaphragmatic hernia and posterior urethral valves

Babies with missing data for principal background variables (gestational age at birth, gender and place of birth) will be excluded.

### Linkage cohorts

**Fig 1:**
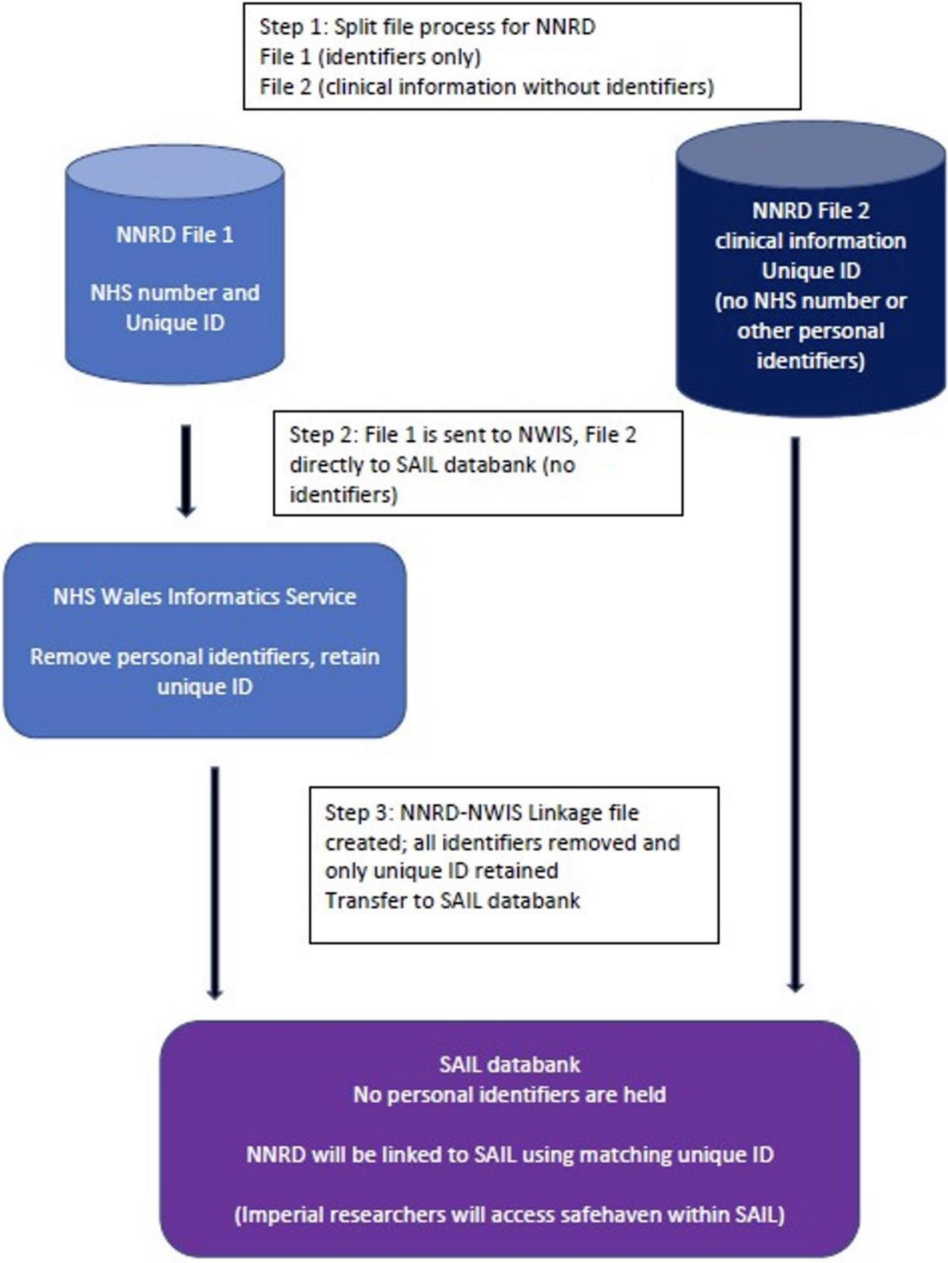
Cohorts for linkage to other health and education databases.

Included children will fall into one or more of four cohorts for linkage to other health and education databases (Fig 1).

- Cohort 1 will include preterm babies (<32 weeks) and babies with surgical conditions of all gestational ages (as listed above), born in England, 2007–2020, for linkage to health data.
- Cohort 2 will include preterm babies (< 32 weeks gestation), born 2007–2016 in England, for linkage to education outcomes.
- Cohort 3 will include babies with surgical conditions of all gestational ages, born 2012–2016 in England, for linkage to education outcomes.
- Cohort 4 will include preterm babies (<32 weeks), born 2012–2020 in Wales for linkage to the SAIL databank (health, education and social data)

Whilst linkage between health datasets will include those born up to 2020, the linkage to English education datasets will be limited to children who have reached school age by the end of 2020 (i.e. born before 2017).

### Recruitment

This study will only use routinely available data and will not actively recruit any patients.

Included children will be identified and data extracted from the Research Ethics Committee-approved National Neonatal Research Database [22].

### Data sources

The final de-identified datasets will be formed by linking together datasets summarised in Table 1. Cohorts will be identified and neonatal data extracted from the National Neonatal Research Database (NNRD) [22]. For children born in England, health data will be sourced from the Office for National Statistics (ONS) [23], Hospital Episode Statistics (HES) [24], the Paediatric Intensive Care Audit Network (PICAnet) [25], and the Mental Health Dataset (MHDS) [26]. Education data will be sourced from the National Pupil Database (NPD) [27]. For children born in Wales, health and education data will be obtained from the Secure Anonymised Data Linkage Data (SAIL) Databank [28].

**Table 1:** Datasets for linkage.

Data source	Description	Summary of data items for extraction
National Neonatal Research Database (NNRD) [22]:	Contains care data for all babies admitted to NHS neonatal units across UK since 2007	Baby’s demographics, neonatal clinical care, diagnoses, outcomes up to neonatal discharge, outcomes at two-year review.
Office for National Statistics (ONS) [23]	Collects data regarding date and cause of death for all registered deaths in England and Wales.	Death registration, including date of death and cause of death.
Hospital Episode for Statistics (HES) [24]	Contains data on hospital admissions to NHS hospitals, outpatient appointments, and attendances at accident and emergency departments across England. Collects data related to patient demographics, diagnoses and clinical care.	Demographics, clinical care and diagnoses.
Secure Anonymised Information Linkage (SAIL) databank [28]	Contains annual district birth and death extracts, congenital anomaly register, education data for Wales, critical care dataset, patient episode database Wales, national community child health database, Welsh demographic service dataset, Welsh longitudinal general practice dataset, Wales results reporting service, maternal indicators dataset.	Demographics, clinical care, diagnoses including congenital anomalies, education, births and deaths, primary and secondary care use, community child health resource use and maternity data.
Paediatric Intensive Care Audit Network (PICANet) [25]	Contains details of the treatment of all critically ill children in paediatric intensive care units (PICU) across the UK. Data includes: demographics; clinical diagnoses; treatment received in PICU and outcomes at discharge.	Demographics, clinical care, diagnoses and outcomes
Mental Health Services Dataset (MHSDS) [26]	The MHSDS contains individual level data for all children accessing mental health care across the community, outpatient, and inpatient settings in England.	Constant supervision and care required due to disability indicator, looked after child indicator, child protection plan indication code, care professional service or team type association (mental health), disability code.
The National Pupil Database (NPD) [27]	Managed and controlled by the Department for Education and held in the ONS Safe Haven. The NPD contains detailed information on the educational attainment, SEN, and attendance of children at state schools across England between the ages of 5–18 years.	The School Census (including alternative provision census and pupil referral unit census, Children in Need, Children Looked After, School Absences, School Exclusions); Early Years Foundation Stage Profile (EYFSP) data at age 5, Phonics data, Attainment, Good for Development (reaching expected attainment), Key Stage 1 data, Key Stage 2 data with attainment scores, Education, health and Care plan (EHC), Special Educational Needs include: primary and secondary SEN type, Disability Access Funding (For 3–4 year olds), eligibility for free school meals.

### Linkage mechanisms: The use of identifiers

Identifiers are necessary to conduct the linkage between the NNRD and other health and education databases. NHS number, date of birth, gender will be used to link the NNRD to English health data (HES, ONS, MHSDS, and PICANet). For Welsh data, the NNRD is linked to SAIL data using NHS number, via Digital Health and Care Wales (previously known as NHS Wales Informatics Service) [29].

Education records do not hold NHS numbers, and therefore forename, surname, date of birth, postcode, gender are required to link the NNRD to the NPD. However, the NNRD does not contain forename, surname or recent postcodes. Thus, the NHS number from NNRD will first be linked to the Personal Demographic Service (PDS) [30] in NHS Digital prior to linkage to the NPD.

### Linkage mechanisms: The split file and third-party linkage process

A “split-file” process will be used to separate personal identifiers from the clinical dataset so that only identifiers (without clinical data) are shared with the independent third party for linkage. These third parties are NHS Digital for English data, and Digital Health and Care Wales for Welsh data.

The data flows are designed such that no organisation will hold data they do not already hold, and researchers will only analyse de-identified data. No clinical data will be transferred to the third parties.

Identifiers will be temporarily used, in a secure environment, for accurate linkage. Once third-party linkage has been carried out, all identifiers (NHS number, forename, surname, date of birth, and postcodes) are removed and the linked records will only retain the anonymised unique ID and clinical/ education data. No researchers will have access to these identifiers.

**Fig 2:**
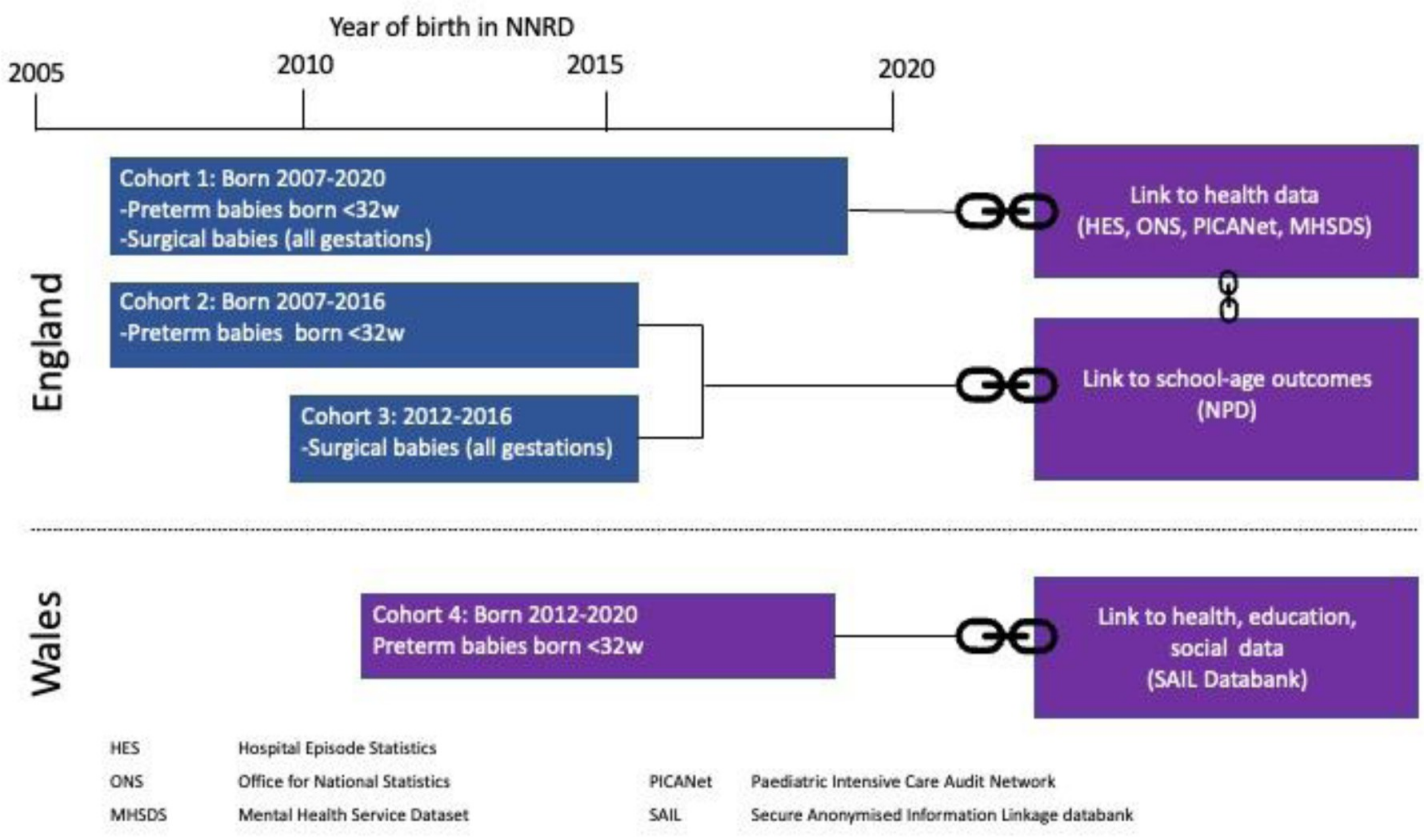
Data flows for the English and Welsh data.

**Fig 3:**
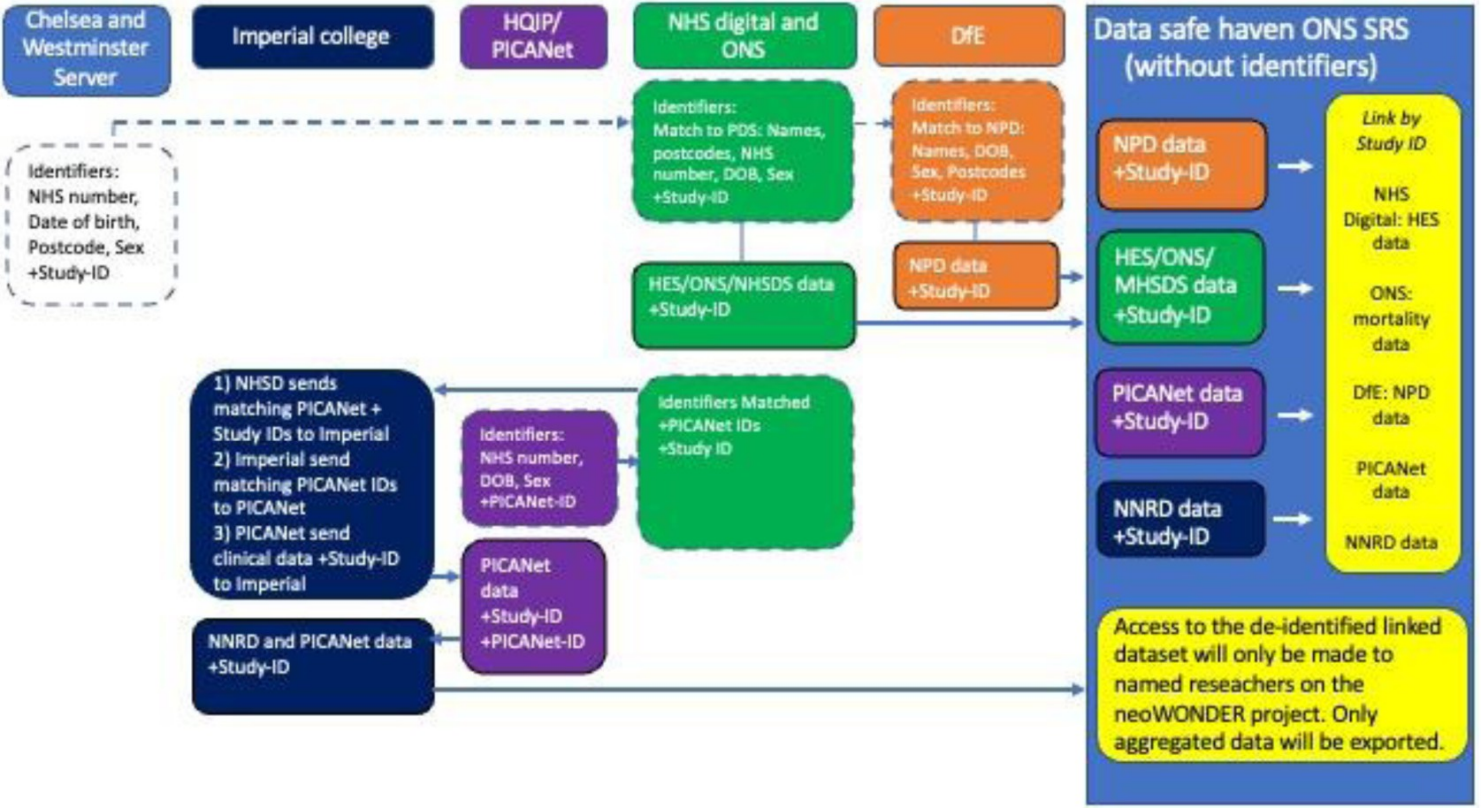
Further data flows for the English and Welsh data.

**Fig 4:**
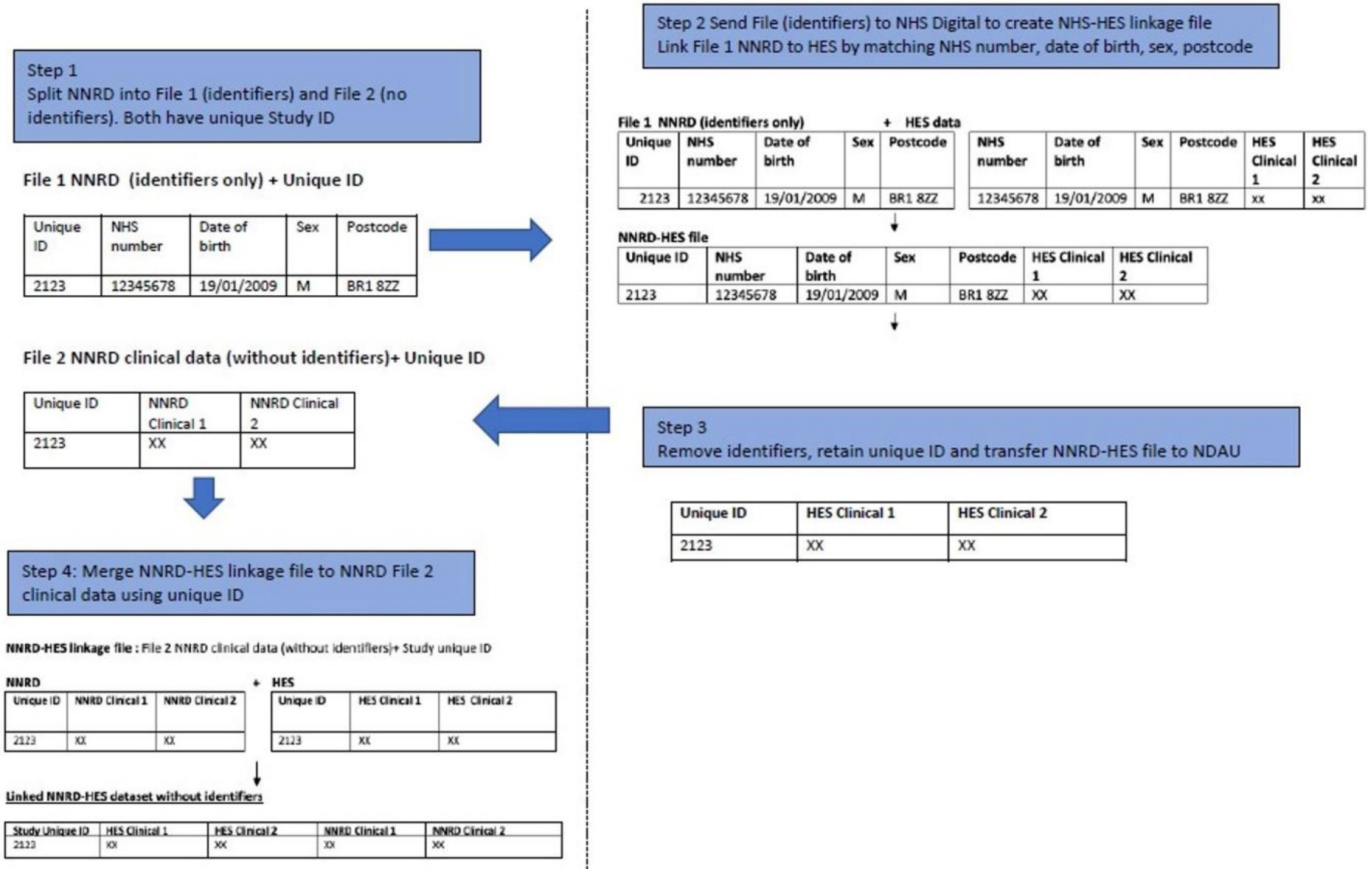
An illustration of the split-file process (using the example of linkage of the NNRD to HES).

The data flows for English and Welsh data are shown in Figs 2 and 3. An illustration of the split-file process (using the example of linkage of the NNRD to HES) is shown in Fig 4. A video for lay audiences explaining the split file process has been produced [31]. Detailed descriptions of the data flows are given in S1 File.

### Data security

Data sharing agreements will be drawn up between data controllers. All data will be transferred securely via approved secure file transfer systems.

The final de-identified linked dataset combining NNRD, HES, ONS, MHDS, PICANet and NPD (without identifiers), will be accessed by researchers in the ONS SRS safe haven [32]. An additional de-identified dataset containing English health data only (NNRD, HES, ONS, MHDS and PICANet) will be held by and accessed through Imperial College London. The final NNRD-SAIL linked dataset will be accessed through the SAIL Databank safe haven, via the SAIL Gateway (a secure remote desktop platform) [33]. Researchers will have undertaken the relevant training courses and been successful in their application to achieve ONS researcher accreditation and/or SAIL Gateway user access.

The Neonatal Data Analysis Unit (NDAU) at Imperial College London will act as the data controller for this study. Imperial College London will keep primary research data for 10 years after the study has completed, in line with Medical Research Council guidance for clinical studies. These data will not contain any identifying information.

### Analysis plan

#### Data linkage rates

Data linkage rates will be examined by applying deterministic (using actual identifier) and probabilistic (if not full complement of identifiers available) linkage. Characteristics of linked and unlinked babies will be compared.

#### Strategies to address potential missing data

Data checks on all data will be completed to investigate missing or implausible data values. Robust standard operating procedures will be developed for handling each variable if appropriate based on level of missingness. If we believe data are missing at random, we will investigate whether multiple imputation may be appropriate. If levels of missing data are high (>10%) for key variables, reasons for this will be investigated, and methods to mitigate the impact on any analysis may be used. If key variables are missing, a sensitivity analysis will be undertaken to impute values. Analyses will be undertaken with and without the babies with missing data to assess the impact on the results.

### How NeoWONDER will address its aims

**Aim one**: **to describe the long-term health and education outcomes for very preterm-born children, and children who have undergone early childhood surgery, and examine factors influencing these outcomes**

**a**. **For very preterm babies born and cared for in England**

The health outcomes of the approximately 102,000 very preterm babies included in cohort one, and the education outcomes for the approximately 80,000 very preterm babies in cohort two will be described. The health outcomes of interest include mental health and behavioural conditions, and health resource use and a health economic evaluation. These outcomes are detailed in S4 File. The education outcomes of interest include school attendance rates, special school attendances rates, statement of educational needs, and educational attainment: at Early Years Foundation Stage (age four years), Key Stage one (age seven years), and Key stage two (age 11 years).

Descriptive analyses will be undertaken on the linked datasets, with important consideration given to data quality and completeness. We will describe the characteristics of admission to neonatal care and subsequent readmissions including length of stay, interventions or procedures and healthcare resource use during the neonatal stay.

We will determine mortality rates and causes of death following discharge, and investigate the risk of admission to paediatric care. We will determine the potential modifying impact of socioeconomic factors on long-term physical health outcomes. To explore the timing of readmission and determine whether there is correlation with diagnoses or demographics, survival analysis approaches such as Cox regression or flexible parametric modelling will be used. The absolute mortality rates and hazard ratios will be reported with 95% confidence intervals. A logistic regression will be undertaken to determine the odds ratio (with 95% confidence intervals) of outcomes of interest (amongst the included population) at predefined ages during the study follow-up period. Subgroup analyses will be undertaken to examine geographic variation, temporal trends, and trends by gender.

We will explore trends in education outcomes (outlined above) for the following subgroups: geographic variation (area of birth); temporal trends (year of birth); eligibility for free school meals; looked after child; first language spoken at home; school type (mainstream or special school); by school ages.

**b**. **For very preterm babies born and cared for in Wales**

Health and education outcomes for the cohort of approximately 3500 very preterm babies born in Wales (cohort four) will be described, and factors influencing these outcomes examined. Descriptive analytics, summary statistics and multivariable logistic regression will be used to determine mortality rates and causes of death following neonatal discharge, health outcomes following discharge (including diagnoses, treatment, medication, and access to primary and secondary health care and procedures), and educational attainment at key stage one, statement of special educational needs, special school, and school attendance. Outcomes will be compared to those of term counterparts born in the same year.

**c**. **For babies with surgical conditions born in England**

The long-term outcomes and academic attainment of the approximately 8000 children who have undergone early childhood surgery will be investigated, and factors associated with educational attainment identified. The primary outcome will be attainment of a Good Level of Development (GLD) on the Early Years Foundation Stage Profile (EYFSP). Secondary outcomes will include mortality, hospital admissions, school absence and special educational needs status. We will perform multiple linear regression for covariates with continuous outcomes and logistic regression for dichotomous outcomes, to analyse the contribution of various determinants on the development of outcomes.

**Aim two**: **to evaluate the impact of neonatal interventions on the later health and educational outcomes of very preterm babies using an exemplar intervention: fortification of human breast milk versus no fortification**

The utility of the linked dataset generated will be tested, aiming to address one of the top priorities for preterm babies, whether fortification of breast milk (versus no fortification) affects key long-term outcomes for very preterm babies.

Only babies who achieve full enteral feeds (defined as three consecutive days with enteral feed given without an intravenous fluid or parenteral nutrition) will be included. Only babies old enough to have an Early Years Foundation Stage Profile (i.e. age five) will be included. Babies with major congenital malformations (likely to influence feeding strategies, and/or require surgery in the neonatal period) will be excluded. Babies who received management for necrotising enterocolitis prior to attaining full enteral feeds will be excluded.

Babies will be grouped by whether they receive breast milk fortifier during their neonatal admission, and those who do not.

The primary outcomes of interest will be attainment of a Good Level of Development on the Early Years Foundation Stage Profile (EYFSP). Secondary outcomes will include: Special Educational Needs provision, diagnosis of developmental delay, diagnosis of Autism, diagnosis of attention deficit hyperactivity disorder, mortality, diagnosis of necrotising enterocolitis requiring surgery or causing death.

Characteristics for the two groups will be described. A propensity model will be developed using a direct acyclic graph to inform the variables included. Missing variables will be described, and strategies developed to address them. Dependant on findings, this may include use of ‘missing’ categories for missing categorical variables, and use of multiple imputation by chained equations. Nearest neighbour matching will be used, with callipers applied to limit the distance between pairs for both specific variables and overall propensity score. Callipers will additionally be set to ensure close matching of the following specific variables: birth year, birth weight decile for gestation, and gestation. For remaining variables, callipers will be applied to achieve absolute standardised mean differences below 0.1. Observations outside the common support will be discarded. The matching ratio will be optimised based on group sizes. Covariate balance will be assessed, with absolute standardised mean differences and Kolmogorov-Smirnov statistics examined for all covariates. Distributions of propensity scores between the two exposure groups were compared before and after adjustment using density estimates. Balance plots will be produced for all variables, using density estimates for continuous variables and histograms for categorical variables. A weighted regression model conditioned on the variables included in matching will be used to give an estimate for each outcome.

### Validation of data for continual improvement in completeness and accuracy of the NNRD

We will provide a feedback validation loop to neonatal units contributing data to the NNRD (the UK Neonatal Collaborative) to validate the NNRD data that will be included in the analyses (detailed in S5 File).

### Ethical considerations and declarations

Research Ethics Committee (REC) approval for NeoWONDER was granted in June 2021 (REC reference 21/EM/0130). Due to the need to access personal identifiable data for the linkage, approvals were obtained from the Confidentiality Advisory Group (CAG) to use identifiers without consent (CAG reference 21/CAG/0081). This enables the linkage to be legally permissible under the Health Research Authority’s support under Section 251 of the NHS Act 2006.

Instructions for how to opt-out of NeoWONDER are detailed on the parent and carer information sheet [34]. Parents can opt-out of their child’s data being used by contacting a member of staff at any neonatal unit where their child received care. To opt-out of their child’s data being transferred to the NNRD, parents can contact the data hosting company, or request that their neonatal unit to do so [35]. For care received in England, parents can also use the National Data Opt-Out [36].

## Discussion

### Limitations

The neoWONDER cohort will be one of the largest longitudinal preterm birth and surgical cohorts curated from linked routine records, providing the statistical power to investigate rare exposures and outcomes. neoWONDER provides whole population coverage for all preterm babies born before 32 weeks’ gestation, and babies of all gestation with key surgical conditions admitted to neonatal units. The whole population nature of the cohort enhances the generalisability of studies using this dataset. However the cohort is restricted to babies admitted to neonatal units in England and Wales and excludes babies who died in the delivery room.

We believe that the use of routinely collected data is an inclusive and cost-effective methodology for addressing questions about long term outcomes. Nevertheless we recognise that certain variables, for example ethnicity, are often inaccurately recorded in routine datasets [37]. The neoWONDER data is derived from routine records, resulting in variable data completeness and accuracy. We will work with hospital coders and teams who record the original data to understand the limitations of the primary data.

The linkage of multiple datasets creates an increased risk of linkage error and potential for linkage bias when the probability of a correct linkage is associated with variables of interest. Missed links may create selection bias and incorrect links may create misclassification bias. Although missed and incorrect linkages will be a property of the neoWONDER dataset, the impact of missed or incorrect linkages will vary as a function of the research question. Each study using the neoWONDER dataset will require the input of statisticians who are knowledgeable about linkage bias to assess its impact.

### Dissemination plans

An animation video explaining data linkage for the lay audience has been produced [31]. Leaflets and written reports summarising the research findings for lay audiences will be produced. These will be disseminated to families through UK neonatal units, the NeoWONDER newsletter and website [34], and the charity BLISS [38] using their established communication channels (newsletters, social media, and volunteers).

Findings will be shared with health professionals and academics through peer-reviewed scientific publications, conference presentations, social media, and a study website.

A written report will summarise the findings for public service providers and policy makers.

## Supporting information

S1 File Detailed description of linkage, data flows and data access by cohort. (DOCX)

S2 File Further detail on NDAU and NNRD. (DOCX)

S3 File Surgical condition codes. (DOCX)

S4 File Health outcomes to be examined for very preterm babies born and cared for in England. (DOCX)

S5 File Validation of data for continual improvement in completeness and accuracy of the NNRD. (DOCX)
