# Supplementary material for: A protocol for neoWONDER: Neonatal whole population data linkage to improve long-term health and wellbeing of preterm and sick babies
Source: PLoS One. 2024 Jul 16;19(7):e0305113. doi: 10.1371/journal.pone.0305113 (PMC11251610; doi:10.1371/journal.pone.0305113)
Supplement: S1 File — (DOCX) [file pone.0305113.s001.docx]

**S1: Detailed description of linkage, data flows and data access by cohort**

### NNRD linkage to HES/ONS/MHSDS: cohort 1

NNRD (File 1 identifiers) will be sent to NHS Digital to be linked to HES, ONS mortality and MHSDS data using the NHS number and date of birth. Identifiers are then moved with retained unique ID and transferred back to the NDAU Imperial College. The unique ID will be used to link File 2 to File 2 at the NDAU. Data will be stored and accessed at NDAU. (Figure 2)

### NNRD linkage to NPD: cohorts 2 and 3

Linkage between the NNRD and NPD requires additional identifiers such as forename, surname and recent postcodes (which are not held on the NNRD). This is because there is no common identifier on both NHS and NPD records. NPD is educational data and holds Unique Pupil Numbers rather than NHS number. As the forename and postcode on the NNRD is likely to have changed by the time the child starts school (4-5 years following discharge from neonatal care), linkage to the Personal Demographic Service (PDS) is necessary to obtain most up to date identifiers to accurately link to the NPD. Hence, File 1 (identifiers) will be transferred to NHS Digital Personal Demographic Service first to link to subsequent postcode addresses following neonatal unit discharge. (Figure 2)

The Department for Education holds the National Pupil Database (NPD) in the Office for National Statistics Secure Research Service (ONS SRS) and data from the NPD cannot leave the ONS SRS. Therefore, identifiers will need to be transferred from PDS to the Department for Education.

The ONS SRS is set up for accredited researchers to access data from the National Pupil Database (NPD). Data cannot be downloaded from the SRS, and ONS procedures ensure it operates within a legal framework without disclosure of sensitive information. Researchers working on this project will obtain the necessary ONS accreditation.

The NNRD file containing identifiers only with unique ID will first be transferred to the NHS Digital PDS. The PDS will use these identifiers to identify additional identifiers such as subsequent postcodes or change of names. Forename, surname, date of birth, gender and postcodes will be securely transferred to the Department for Education to be used to link to educational data within the NPD. A logic model, designed to maximize the chance of a reliable postcode match (given the variation over time), will be used. This is an established model developed to improve the linkage of health and NPD data. The quality of linkage will be evaluated with the data controllers and un-linked records will be reviewed and the sensitivity of the probabilistic matching algorithms maximised. NDAU will then send clinical data and unique ID (without identifiers) to ONS SRS securely and link to NPD (without identifiers) using unique ID.

Data will be accessed within the ONS SRS.

### NNRD to SAIL databank (Wales): cohort 4

Step 1: NDAU send File 1 (personal identifiers only) to The NHS Wales Informatics Service

(NWIS) third party for linkage to SAIL databank

Step 2: NDAU send File 2 (NNRD clinical data with unique ID and no identifiers) by secure transfer to SAIL databank to link Safehaven for final linked dataset: SAIL databank, which does not hold any identifiers.

Data will be accessed through the SAIL Databank.
