# Supplementary material for: A protocol for neoWONDER: Neonatal whole population data linkage to improve long-term health and wellbeing of preterm and sick babies
Source: PLoS One. 2024 Jul 16;19(7):e0305113. doi: 10.1371/journal.pone.0305113 (PMC11251610; doi:10.1371/journal.pone.0305113)
Supplement: S2 File — (DOCX) [file pone.0305113.s002.docx]

**S2: Further detail on NDAU and NNRD**

The Neonatal Data Analysis Unit (the NDAU) is a research unit in the Faculty of Medicine, Imperial College London and is based at Chelsea and Westminster Hospital. The NDAU was established to improve the quality of operational clinical data captured at the point of clinical care and promote their best use to support neonatal services and facilitate research. The NDAU is led by a multi-professional Steering Board that includes parent and patient representatives. NDAU complies with the National Statistics Practice and Protocol on Data Access and Confidentiality.

The Neonatal Data Analysis Unit (NDAU) at Imperial College is the data controller of the NNRD. NDAU have a robust information governance framework for information management and have deployed a range of privacy enhancing technologies, physical security measures and audit procedures to safeguard the data according to rigorous standards.

The NNRD is formed from clinical data about mother and baby that are extracted from routinely recorded NHS care records. Data recorded are securely transferred to servers based within the Chelsea and Westminster NHS Foundation trust. Following receipt of these data, any personal information (for example NHS numbers) are removed and held separately on the Chelsea and Westminster server. The de-identified data (without personal information) is transferred to Imperial College server.
