# Supplementary material for: A protocol for neoWONDER: Neonatal whole population data linkage to improve long-term health and wellbeing of preterm and sick babies
Source: PLoS One. 2024 Jul 16;19(7):e0305113. doi: 10.1371/journal.pone.0305113 (PMC11251610; doi:10.1371/journal.pone.0305113)
Supplement: S3 File — (DOCX) [file pone.0305113.s003.docx]

**S3: Surgical conditions definitions**

Surgical conditions are identified from the NNRD using the following definitions.

1. Necrotising Enterocolitis

The following terms in PrincipalDiagnosisAtDischarge, GastrointestinalDiagnoses or PrincipleProceduresDuringStay:

- NECROTISING ENTEROCOLITIS
- NECROTISING ENTEROCOLITIS – PERFORATED
- NECROTISING ENTEROCOLITIS - PROVEN (ON XRAY OR AT SURGERY)
- NECROTISING ENTEROCOLITIS – CONFIRMED
- LAPAROTOMY
- LAPAROTOMY APPROACH NEC
- COLECTOMY AND ILEOSTOMY NEC

AND

The following in the daily table:

NECTreatment = 2 (surgical)

THEN surgical NEC = 1

OR

The following in the abdominal x-ray table:

LaparotomyPerformed = ‘y’ and HistologyConfirmationNEC = ‘Yes’

1. Hirschsprung’s disease

The following terms in PrincipalDiagnosisAtDischarge, GastrointestinalDiagnoses, PrincipleProceduresDuringStay or diagnosis table:

- HIRSCHSPRUNG’S DISEASE LONG-SEGMENT: 16227, 10501
- HIRSCHSPRUNG’S DISEASE SHORT-SEGMENT: 16228, 10502
- HIRSCHSPRUNG’S ENTEROCOLITIS: 10503
- PULL THROUGH FOR HIRSCHSPRUNGS DISEASE (ANY TECHNIQUE): 100039
- DEFINITIVE HIRSCHSPRUNGS SURGERY DUHAMEL: 11270
- DEFINITIVE HIRSCHSPRUNGS SURGERY LESTER MARTIN
- DEFINITIVE HIRSCHSPRUNGS SURGERY TRANSANAL PULL THROUGH: 11272
- DEFINITIVE HIRSCHSPRUNGS SURGERY REHBEIN
- DEFINITIVE HIRSCHSPRUNGS SURGERY SOAVE-BOLEY: 11274
- LAPAROSCOPIC ASSISTED DEFINITIVE HIRSCHSPRUNGS SURGERY: 11499
- HIRSCHSPRUNGS DISEASE: 16226, 16227
- TOTAL INTESTINAL AGANGLIONOSIS: 11058
- EXTENDED RIGHT HEMICOLECTOMY WITH ILEOSTOMY MUCUS FISTULA

1. Gastroschisis

The following terms in PrincipalDiagnosisAtDischarge, GastrointestinalDiagnoses, PrincipleProceduresDuringStay or diagnosis table:

- DELAYED CLOSURE GASTROSCHISIS: 11277
- PRIMARY REPAIR OF GASTROSCHISIS: 11654
- REPAIR GASTROSCHISIS USING PROSTHESIS: 11708
- SILO INSERTION FOR REDUCTION OF GASTROSCHISIS: 11784
- CLOSURE OF GASTROSCHISIS INCLUDES CLOSURE OF EXOMPHALOS: 1006730
- GASTROSCHISIS: 16499

1. Oesophageal Atresia

The following terms PrincipalDiagnosisAtDischarge, GastrointestinalDiagnoses, PrincipleProceduresDuringStay or diagnosis table:

- ESOPHAGEAL ATRESIA
- OESOPHAGEAL ATRESIA WITH DISTAL TRACHEA-OESOPHAGEAL FISTULA: 10740
- OESOPHAGEAL ATRESIA WITHOUT DISTAL FISTULA
- PRIMARY REPAIR OF OESOPHAGEAL ATRESIA: 11658
- OESOPHAGEAL ATRESIA WITHOUT TRACHEOESOPHAGEAL FISTULA: 10741, 010245
- OESOPHAGEAL ATRESIA WITH TRACHEOESOPHAGEAL FISTULA: 10740, 1010246
- ATRESIA OF OESOPHAGUS WITHOUT FISTULA: 16195
- ATRESIA OF OESOPHAGUS WITH TRACHEA-OESOPHAGEAL FISTULA: 16196
- TRACHEOESOPHAGEAL ATRESIA AND/OR/FISTULA REPAIR: 100010
- OESOPHAGEAL ATRESIA WITHOUT TRACHEOESOPHAGEAL FISTULA: 1010245, 10741
- ESOPHAGEAL ATRESIA

1. Congenital Diaphragmatic Hernia

The following terms in PrincipalDiagnosisAtDischarge, GastrointestinalDiagnoses, PrincipleProceduresDuringStay or diagnosis table:

- CONGENITAL DIAPHRAGMATIC HERNIA: 10246
- MORGAGNI DIAPHRAGMATIC HERNIA: 10694
- RECURRENT CONGENITAL DIAPHRAGMATIC HERNIA: 10905
- OTHER REPAIR OF DIAPHRAGMATIC HERNIA (SPECIFY): 11597
- CONGENITAL DIAPHRAGMATIC HERNIA: 10246
- PRIMARY REPAIR OF CONGENITAL DIAPHRAGMATIC HERNIA: 11657
- PROSTHETIC REPAIR OF CONGENITAL DIAPHRAGMATIC HERNIA: 11660
- FETOSCOPIC INSERTION OF TRACHEAL PLUG FOR CONGENITAL DIAPHRAGMATIC HERNIA: 1006132
- PERCUTANEOUS INSERTION OF TRACHEAL PLUG FOR CONGENITAL DIAPHRAGMATIC HERNIA
- REPAIR OF CONGENITAL DIAPHRAGMATIC HERNIA: 1006671
- REPAIR OF DIAPHRAGMATIC HERNIA
- OTHER SPECIFIC REPAIR OF DIAPHRAGMATIC HERNIA: 1001924
- REPAIR OF DIAPHRAGMATIC HERNIA USING ABDOMINAL APPROACH: 1001923
- REPAIR OF DIAPHRAGMATIC HERNIA USING THORACIC APPROACH
- UNSPECIFIED REPAIR OF DIAPHRAGMATIC HERNIA: 1001925

1. Posterior Urethral Valves

The following terms in PrincipalDiagnosisAtDischarge, GastrointestinalDiagnoses, PrincipleProceduresDuringStay or diagnosis table:

- POSTERIOR URETHRAL VALVES: 10854
- ENDOSCOPIC DESTRUCTION OF URETHRAL VALVES: 1004687
- HOOK ABLATION OF POSTERIOR URETHRAL VALVE: 11444
- CONGENITAL POSTERIOR URETHRAL VALVES: 10854, 16357
- RESECTION OF POSTERIOR URETHRAL VALVES: 100058
- ENDOSCOPIC RESECTION OF POSTERIOR URETHRAL VALVE: 11338
