# Supplementary material for: A protocol for neoWONDER: Neonatal whole population data linkage to improve long-term health and wellbeing of preterm and sick babies
Source: PLoS One. 2024 Jul 16;19(7):e0305113. doi: 10.1371/journal.pone.0305113 (PMC11251610; doi:10.1371/journal.pone.0305113)
Supplement: S4 File — (DOCX) [file pone.0305113.s004.docx]

**S4: Health outcomes to be examined for very preterm babies born and cared for in England**

- Short term outcomes during the neonatal unit prior to discharge will be examined as these are intermediate outcomes that influence longer term outcomes. These include diagnoses (e.g. congenital anomalies, brain injury, necrotising enterocolitis, retinopathy of prematurity, bronchopulmonary dysplasia) and outcomes (e.g. survived to discharge from neonatal care, two-year neurodevelopmental outcomes)
- All-cause mortality
- Cause-specific mortality
- Mental health and behavioural conditions. The first diagnosis of any of the following conditions (identified with predefined ICD 10/SNOMED codes): conduct disorder, emotional disorders such as depression and anxiety, hyperactivity disorders e.g. ADHD, social and communication disorders e.g. Autism Spectrum Disorders
- Chronic conditions. The first diagnosis of any of the following conditions (using ICD 10/SNOMED codes): including cancer and blood conditions, cardiovascular, respiratory conditions, musculoskeletal/ dermatological conditions, neurological conditions, metabolic/ endocrine/ digestive conditions, renal/ genitourinary conditions)
- Health resource use: frequency of visits, use of secondary care and Paediatric intensive care, length of stay
- Number and proportion Incidence of CAMHS referrals and Prevalence of mental health problems (who accessed CAMHS services, with mental health diagnoses, accessing secondary health services)
- Health economic evaluation including health and educational resource needs
