# Supplementary material for: A protocol for neoWONDER: Neonatal whole population data linkage to improve long-term health and wellbeing of preterm and sick babies
Source: PLoS One. 2024 Jul 16;19(7):e0305113. doi: 10.1371/journal.pone.0305113 (PMC11251610; doi:10.1371/journal.pone.0305113)
Supplement: S5 File — (DOCX) [file pone.0305113.s005.docx]

**S5: Validation of data for continual improvement in completeness and accuracy of the NNRD**

Optimising completeness and accuracy of routinely recorded data held on the NNRD will improve the validity of the study findings. This is particularly important for workstream 3 when the impact of neonatal interventions on outcomes are evaluated. Core neonatal outcomes held on the NNRD (such as brain injury, necrotising enterocolitis, spontaneous intestinal perforation, sepsis, bronchopulmonary dysplasia, retinopathy of prematurity, two-year neurodevelopmental outcomes) are important short and intermediary outcomes that are included in the analyses as they influence longer term outcomes in later life.

We will provide a feedback validation loop to neonatal units contributing data to the NNRD (the UK Neonatal Collaborative) to validate the NNRD data that will be included in the analyses.

We will contact neonatal units by secure nhs.net email address to involve them in data validation for specific studies. Units will have the opportunity to opt-out of participating. The steps will involve:

1. Using secure NHS.net email, a spreadsheet including the BadgerID (pseudonymised local ID) and information held on the NNRD will be sent to the local clinician. The BadgerID are local IDs are only meaningful to local clinicians and cannot be used to link to any other datasets and are not personal identifiers. They will be used by the local clinicians to identify the babies on their local electronic patient record (EPR) system. No patient identifiers will be shared.
2. The local clinician will confirm on the spreadsheet whether the existing diagnoses are correct or whether any need to be changed on the EPR system.
3. The clinicians will return the spreadsheet, containing the validated diagnoses, by secure NHS.net email to the clinical research team
4. The local team will revise inaccuracies in the local electronic patient record system, so that the National Neonatal Research Database is corrected at the next data upload. This will ensure continual improvement in data quality held on the NNRD.
